# FAM134B in Cellular Homeostasis: Bridging Endoplasmic Reticulum-Phagy to Human Diseases

**DOI:** 10.7150/ijbs.113890

**Published:** 2025-08-30

**Authors:** Ning Chen, Jia-Qi Yang, Sen Tong, Lu Xu, Ning Dong, Yao Wu, Yu-Xuan Li, Ren-Qi Yao, Yong-Ming Yao

**Affiliations:** 1 Medical Innovation Research Division and Fourth Medical Center, Chinese PLA General Hospital, Beijing 100853, China.; 2 Department of Acute Abdominal Surgery, Beijing Chao-Yang Hospital, Capital Medical University, Beijing 100020, China.; 3 Department of General Surgery, First Medical Center of the Chinese PLA General Hospital, Beijing 100853, China.; Ning Chen and Jia-Qi Yang have contributed equally to this work.

**Keywords:** FAM134B, ER-phagy, cellular homeostasis, human diseases

## Abstract

FAM134B, originally characterized as an oncogene in esophageal squamous carcinoma, has also been implicated in the pathogenesis of hereditary sensory and autonomic neuropathy type IIB (HSAN2B). It is recognized as the inaugural endoplasmic reticulum (ER)-phagy receptor in mammals containing an LC3-interacting region, which facilitates its interaction with LC3 and GABARAP proteins in the autophagosome. ER-phagy, a critical process involved in ER quality control, selectively degrades superfluous or damaged ER fragments in lysosomes, thereby maintaining ER and protein homeostasis. This review offers an in-depth analysis of FAM134B's structure, function, and regulation, emphasizing its role in infectious diseases, neuropathies, cancer, metabolic disorders, degenerative conditions, and cardiovascular diseases. The evidence presented highlights the need for further research on FAM134B as a potential therapeutic target in human diseases.

## Introduction

Autophagy, or macroautophagy, is a conserved cellular process in eukaryotes that employs lysosomes to degrade cytoplasmic proteins and damaged organelles. Autophagy-related genes regulate the formation of autophagic vesicles for substrate sequestration and autolysosomes for degradation. Under normal conditions, autophagy operates at a baseline level, however, it undergoes significant alterations in response to cellular stress 1. This dynamic process is integral to the synthesis, breakdown, and recycling of cellular components, thereby sustaining homeostasis within the cell. However, excessive autophagy can lead to metabolic stress, cellular degradation, and cell death 2. Recent studies have shown that autophagy, once considered a non-selective process, can specifically target certain cellular components such as impaired organelles, aggregated proteins, and invasive bacteria 3, 4. ER-phagy, a form of selective autophagy, targets excess or damaged ER for degradation within lysosomes or vacuoles. The ER, a single-membrane intracellular system, is essential for protein synthesis, calcium regulation, and lipid metabolism. Thus, ER-phagy is essential for ER quality control and overall cellular homeostasis. Three distinct ER-phagy pathways have been identified: macro-ER-phagy, micro-ER-phagy, and LC3-dependent vesicular transport. To date, reported ER-phagy receptors include membrane-bound ER-phagy receptors (FAM134 protein family, RTN3L, SEC62, CCPG1, TEX264, atlastin (ATL) GTPases, STING) and cytoplasmic ER-phagy receptors (CALCOCO1, C53, p62) 5. Notably, FAM134B was the first ER-phagy receptor discovered in mammals, exhibiting functional homology with Atg40, an ER receptor concurrently reported in yeast 6.

During ER-phagy, FAM134B strongly interacts with MAP1LC3B and GABARAP-L2. Enrichment analysis has revealed that FAM134B interacts with a diverse array of proteins involved in critical cellular functions, including ER function, ATP synthesis, translocon complexes, ER morphology, vesicle formation, Ca^2+^ signaling, and cytoskeletal microfilaments. Additionally, FAM134B is linked to various organelles, including ubiquitin (Ub)-proteasome system, mitochondria, and Golgi apparatus 7. Downregulation of FAM134B leads to ER and Golgi expansion, while its overexpression leads to ER fragmentation and lysosomal degradation in human cells 8. Beyond its role in selective ER-phagy, FAM134B also facilitates LC3B-dependent vesicle translocation to degrade misfolded polypeptide aggregates. We undertook a thorough search and review of the published literature to elucidate the significant biological functions of FAM134B and to offer theoretical guidance for future research endeavors.

## Review Methodology

A literature search on PubMed and Web of Science covered original research in peer-reviewed journals from 2010 to 2024. Studies were chosen for their relevance to FAM134B mechanisms or diseases, excluding conference abstracts and non-peer-reviewed works. The search terms applied in our study included "FAM134B" or "RETREG1" as well as "ER-Phagy" or "reticulophagy".

### Structure and expression patterns of FAM134B proteins

**Figure 1:**
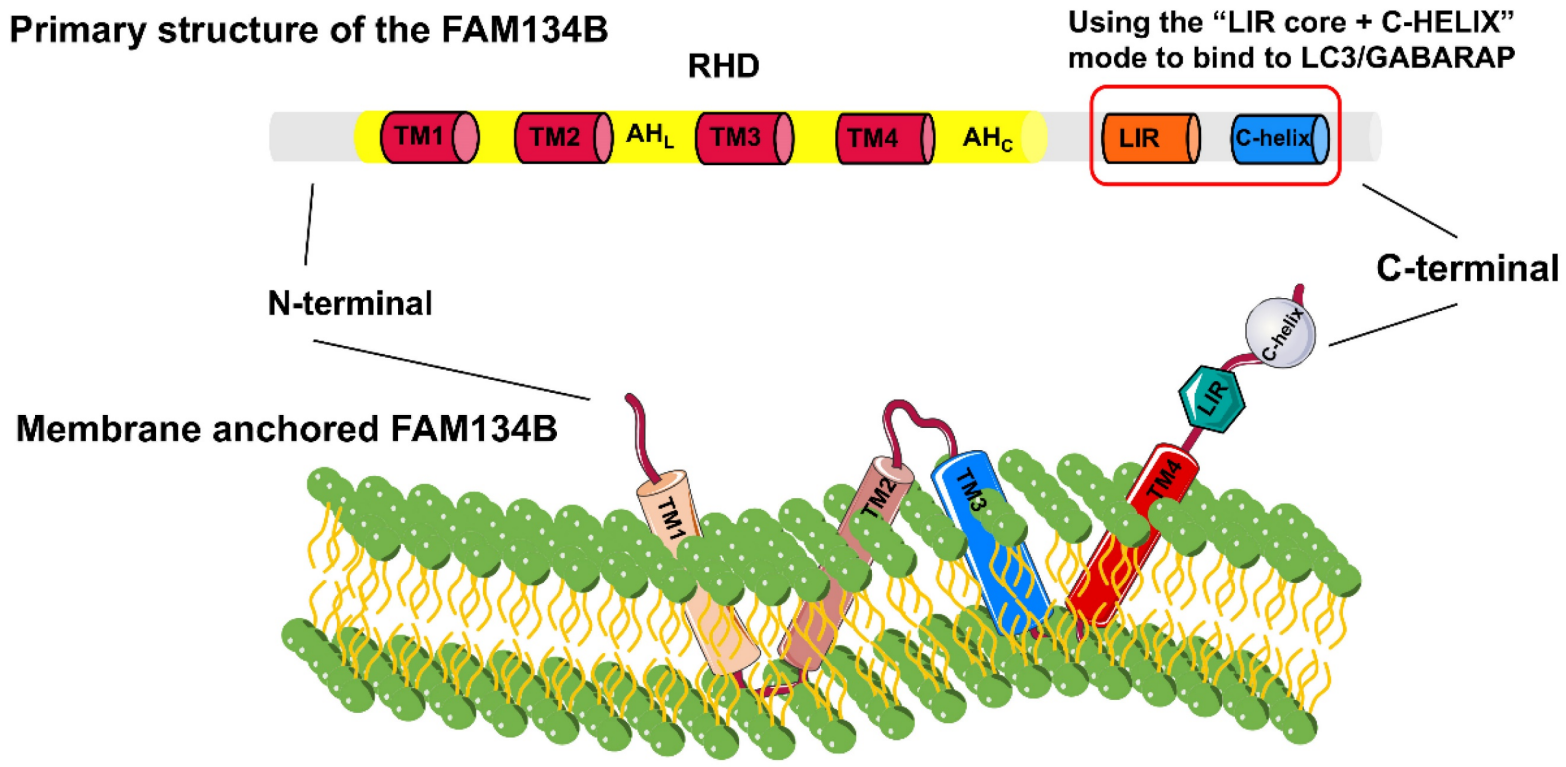
** Detailed structural composition of FAM134B.** FAM134B is an ER-phagy receptor found in *Homo sapiens* and other mammals. The structure of FAM134B consists of three main components: the N-terminal region, reticulon homology domain (RHD), and C-terminal region. The RHD contains TM1-2, AH_L_, TM3-4, and AH_C_, which are primarily responsible for detecting and promoting membrane curvature and scission. The C-terminus contains an LC3-interacting region (LIR) motif.

The FAM134 family, comprising FAM134A/RETREG2, FAM134B/RETREG1, and FAM134C/RETREG3, is crucial for ER fragmentation and lysosomal degradation during ER stress 9. The *FAM134B* gene (also known as *RETREG1* or *JK-1*) is located on chromosome 5p15.1 and spans approximately 144 kb. FAM134B is a 497-amino acid protein with a molecular weight of 54 kDa, primarily located in the ER sheet and Golgi apparatus. Structurally, FAM134B comprises three domains (**Fig. 1**): a N-terminal disordered domain (~80 amino acids), a reticulon homology domain (RHD), and a C-terminal disordered domain (~240 amino acids) containing an LC3-interacting region (LIR) motif 6, 10-12. The LIR motif-mediated interaction between FAM134B and LC3/GABARAP is essential for targeting ER fragments to autophagosomes, leading to subsequent lysosomal degradation 8. The RHD of FAM134B plays a fundamental role in ER membrane remodeling and consists of four key structural components: TM1-TM2, AH_L_, TM3-TM4, and AH_C_ 13, 14. TM1-TM2 and TM3-TM4 are two helical hairpins, each containing two transmembrane (TM) helices linked by a polar luminal loop, which is crucial for membrane deformation and protein clustering. AH_L_ and AHc, two cytoplasmic amphipathic helices, flank TM3-TM4, linking TM1-TM2 with TM3-TM4 and TM3-TM4 with the C-terminus of FAM134B, respectively. These interactions are essential for membrane shaping. The asymmetric wedge structure of RETREG1 RHD facilitates ER membrane bending, whereas the aggregation of six RHDs forms an inverted pyramid structure, further enhancing membrane curvature. During ER-phagy, the clustering of FAM134B RHDs, combined with the pulling force exerted by the C-terminal LIR motif binding to LC3 on the phagophore, promotes vesicle budding, ER pinching off, and subsequent lysosomal degradation 15.

FAM134B-2, a truncated isoform of FAM134B, partially lacks the RHD and consists of six exons encoding a 356-amino acid protein. Starvation leads to an increase in FAM134B-2 expression rather than FAM134B-1 in the liver. Starvation activates the C/EBPβ-FAM134B-2 axis, initiating ER-phagy and lysosomal degradation of FAM134B-2. The ER secretory protein ApoCIII interacts with FAM134B-2 and is degraded through FAM134B-2-mediated ER-phagy, without affecting overall ER turnover 16. Moreover, amino acid deprivation (AAD) in HeLa cells activates the transcription factors MEF2D and NR4A1, which bind upstream of the *FAM134B-2* promoter to enhance mRNA expression and facilitate FAM134B-2-mediated ER-phagy, thereby maintaining amino acid homeostasis. MEF2D functions upstream of NR4A1, and the MEF2D-NR4A1-FAM134B2-mediated ER-phagy cascade is Ca^2+^- and PKC-dependent; however, the precise PKC-dependent mechanisms remain to be elucidated 17. Within the FAM134 family, the lysosomal flux of FAM134B shows a greater increase in BafA-treated U2OS cells compared to FAM134A and FAM134C 18. The wedge-shaped RHD of FAM134B allows for quicker ER vesicle formation compared to FAM134A and FAM134C, enhancing ER membrane remodeling efficiency 9.

### Biological functions and regulatory mechanisms of FAM134B

#### FAM134B and ER-phagy

Under stressful conditions, misfolded protein accumulation within the ER disrupts cellular function. To restore cellular homeostasis, cells initiate ER-phagy, a selective autophagic process that eliminates excess or misfolded proteins and damaged ER membranes. ER-phagy is driven by specific receptors and ultimately integrates into different stages of classical autophagy (recognition, encapsulation, fusion, and degradation) to achieve targeted degradation of abnormal ER components. So far, the dual-fluorescence reporter system (e.g., ssRFP-GFP-KDEL or mCherry-GFP-FAM134B) is the primary method for monitoring ER-phagy flux, leveraging GFP quenching in lysosomal acidity while RFP/mCherry remains stable, allowing for quantification via fluorescence co-localization or ratio changes 19. FAM134B is critical for maintaining physiological homeostasis and modulating pathological conditions by regulating ER dynamics 8. During ER-phagy, FAM134B's LIR domain interacts with LC3-like proteins within the autophagosome and is subsequently transported to the lysosome for degradation through core autophagy machinery. Disruptions in FAM134B function result in significant ER expansion, whereas upregulated FAM134B leads to ER fragmentation.

#### FAM134B and ER-mitochondria interaction

Calcium homeostasis is maintained through the interaction between the ER and mitochondria. Excessive mitochondrial calcium accumulation triggers mitochondrial dysfunction and neuronal apoptosis, representing a significant pathogenic mechanism in acquired epilepsy. The FAM134B-mediated ER-phagy could mitigate mitochondrial calcium overload by diminishing specific receptor IP3R levels on the mitochondrial-associated ER membranes. This action provides a protective effect on mitochondria and reduces aberrant neuronal cell death in the context of acquired epilepsy 20, 21. Beyond its role in Ca²⁺ regulation, FAM134B is involved in the turnover of dual organelles, the ER and mitochondria, to maintain cellular homeostasis. Elevated cytosolic Ca^2+^ levels induce the upregulation of E3 Ub ligase autocrine motility factor receptor (AMFR), which facilitates the proteasomal degradation of mitofusin on the outer mitochondrial membrane under stress conditions. This process results in the formation of unstable mitoplasts, exposing the inner mitochondrial membrane (IMM) and promoting closer ER-mitochondria interactions. The interaction between FAM134B and optic atrophy 1, a mitochondrial dynamin-like GTPase located in the IMM, triggers “reticulo-mito-phagy”, wherein autophagic vesicles encapsulate AMFR and IMM components, targeting them for lysosomal degradation. Reticulo-mito-phagy serves as a protective mechanism that regulates ER-mitochondria crosstalk and restores cellular homeostasis 22. Additionally, FAM134B facilitates adipocyte differentiation by promoting mitophagy and reducing mitochondrial numbers, further underscoring its role in cellular metabolism 23.

#### FAM134B mediated ERLAD

In contrast to the double-membrane autophagosomes in ER-phagy, ER-to-lysosome-associated degradation (ERLAD) involves the transport of a single-membrane vesicle from ER to lysosome. The process of ERLAD requires the interaction between FAM134B and LC3, along with LC3 lipidation, which is essential for vesicle trafficking. Proteasome-resistant polymers of ATZ, a mutant alpha-1-antitrypsin (AAT) protein, accumulate in the ER lumen and are segregated by calnexin (CNX) into a FAM134B-enriched ER subdomain. Subsequently, FAM134B facilitates LC3II-dependent vesicle translocation, encapsulating CNX-FAM134B-LC3II complexes containing ATZ_NNN_ polymers within SNARE STX17 ER vesicles. These vesicles then migrate to LAMP1/RAB7-positive endolysosomes, where they facilitate ATZ polymer degradation 24. Misfolded proteins within the ER are degraded via mannose and glucose processing of N-glycans, which activate either the ER-associated degradation (ERAD) pathway or the ERLAD pathway, respectively. In parallel with classical ER-phagy, FAM134B can mediate ERLAD independently of autophagic vesicle formation. Proteasome-resistant ATZ polymers, which undergo N-glycan processing, generate ERAD-resistant ATZ_NNN_ polymers that interact with CNX and are selectively segregated into the FAM134B-enriched ER subdomain. This process is independent of ER-phagy, but the LIR function of FAM134B and CNX's glucose-binding activity are essential for endolysosomal clearance 24, 25.

#### Regulatory mechanisms of FAM134B expression

Transcription factors, including TFEB/TFE3, activate FAM134B-mediated ER-phagy by inducing FAM134B expression during prolonged starvation. Dephosphorylated TFEB/TFE3 translocates to the nucleus and binds specific DNA-binding site to enhance FAM134B transcription 26, 27. In maintaining ER morphology, the ATL protein family—consisting of ATL1, ATL2, and ATL3—likely exerts complementary functions in regulating ER-phagy. Their GTPase domains aid in ER membrane scission for lysosomal engulfment. FAM134B upregulation results in decreased ATL2 levels, whereas ATL2 downregulation diminishes FAM134B-mediated ER-phagy triggered by FAM134B overexpression. Therefore, ATL2 operates downstream of FAM134B, serving as a FAM134B-mediated ER-phagy effector and cargo 28. Similarly, DDRGK domain-containing 1 acts as a cargo in FAM134B-mediated ER-phagy, with its depletion hindering ER-phagy triggered by FAM134B overexpression, regardless of starvation conditions 29.

The nucleotide exchange factor SIL1, a co-chaperone of BiP, enhances BiP's ATPase activity and energy exchange efficiency in molecular chaperone dynamics. Pathogenic SIL1 mutations impair BiP-mediated protein folding, significantly reducing FAM134B expression. Notably, SIL1 and FAM134B colocalize in the Golgi apparatus 30. Additionally, N6-methyladenosine (m6A) mRNA modification regulates FAM134B expression and affects fat formation in porcine adipocytes. YTH m6A RNA-binding protein 2 (YTHDF2) binds to the m6A site of FAM134B, promoting mRNA degradation and lowering FAM134B levels. A mutation at the m6A binding site prevents mRNA degradation, leading to increased levels of downstream proteins, which boost fat deposition 31.

**Figure 2:**
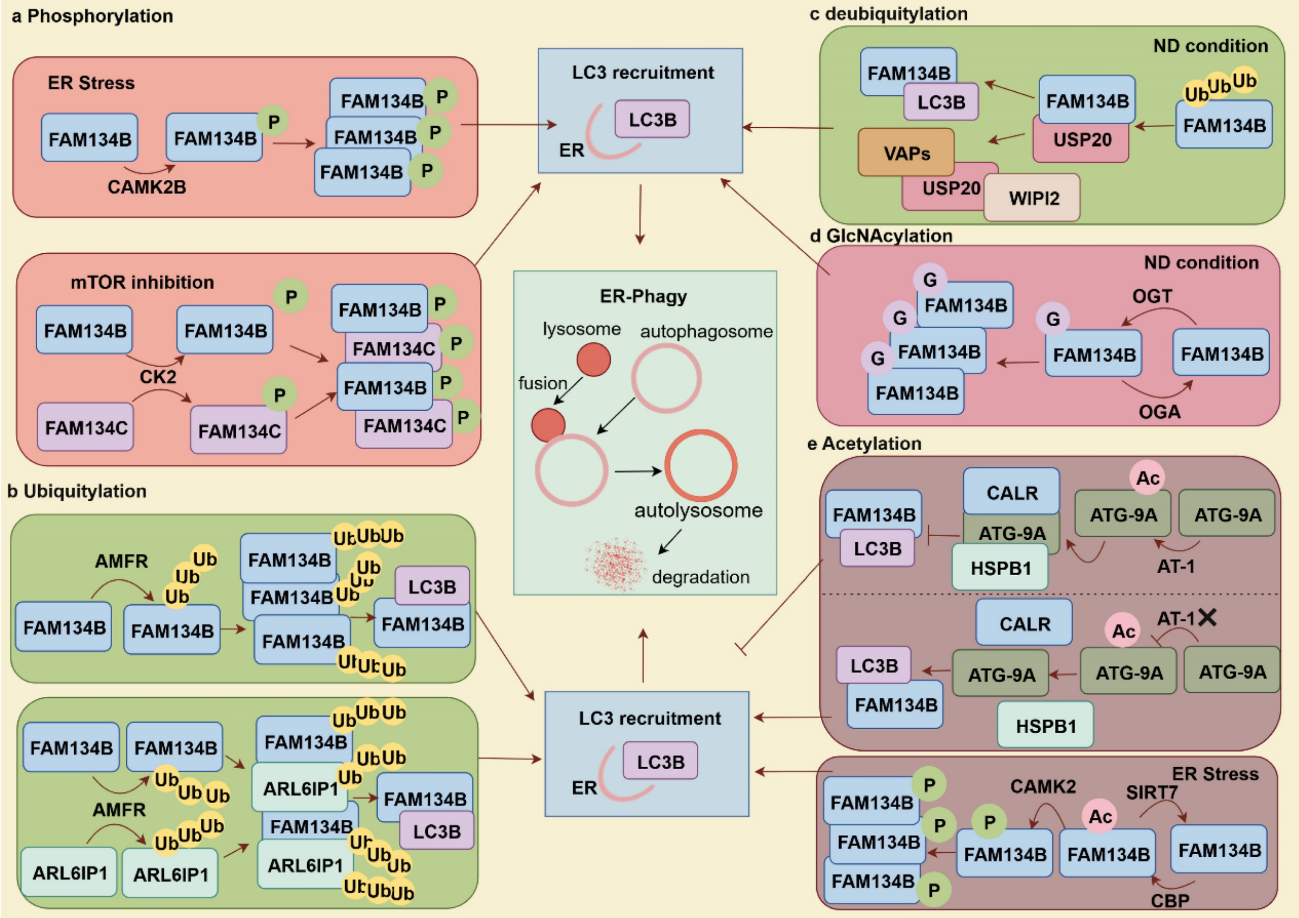
** Post-translational modifications of FAM134B promote or inhibit ER-phagy.** (a) Phosphorylation of FAM134B enhances ER-phagy by promoting its oligomerization and ER fragmentation. FAM134C acts as a substrate and an enhancer for FAM134B-mediated ER-phagy. (b) Ubiquitination of FAM134B and ARL6IP1 by AMFR enhances FAM134B oligomerization and facilitates the formation of FAM134B/ARL6IP1 heterodimers, thereby promoting ER-phagy. (c) USP20 specifically deubiquitinated FAM134B by K48/K63-linked polyubiquitination, activating ER-phagy during starvation. USP20 also interacts with VAPs, facilitating the recruitment of autophagy initiation proteins, such as WIPI2. (d) Under nutrient deprivation conditions, the O-GlcNAcylation of FAM134B by OGT facilitates ER-phagy and cell survival. (e) The acetylated ATG-9A regulates FAM134B-mediated ER-phagy. AT-1/SLC33A1 mediated acetylation of ATG-9A enhances its interaction with the chaperones HSPB1 (cytoplasmic) and CALR (ER lumen), preventing its binding to FAM134B and SEC62, thereby inhibiting ER-phagy. Conversely, AT-1/SLC33A1 knockdown enhances ER-phagy. The acetyltransferase CBP and deacetylase SIRT7 are critically involved in modulating the acetylation status of FAM134B, with acetylated FAM134B facilitating CAMK2-mediated phosphorylation, leading to increased ER-phagy.

Post-translational modifications of autophagy-related factors can either activate or inhibit autophagy, thereby influencing disease progression and drug efficacy (**Table 1**) 32. FAM134B and ER-phagy are regulated by phosphorylation, ubiquitination, O-GlcNAcylation, and acetylation (**Fig. 2**). Phosphorylation of FAM134B sequentially induces oligomerization, ER fragmentation, and ER-phagy. During ER stress, CAMK2B phosphorylation modifies the S151 site of the RHD in FAM134B, promoting FAM134B-mediated ER-phagy to maintain cellular homeostasis 33, 34. Similarly, mTOR inhibition triggers casein kinase 2 (CK2) to phosphorylate FAM134B and FAM134C, leading to their CK2-dependent ubiquitination. Phosphorylation promotes the growth and densification of FAM134 clusters, triggering ER-phagy once these high-density nanoscale clusters reach 90-100 nm. Notably, FAM134C functions as a substrate and an enhancer of FAM134B-mediated ER-phagy 35.

Protein ubiquitination is the tagging of target proteins for degradation, involving ubiquitin activation (E1), conjugation (E2) and ligation (E3) 36. AMFR, an E3 ligase catalyzes the FAM134B's RHD ubiquitination, promoting FAM134B oligomerization and inducing membrane curvature, which enhances ER-phagy flux by facilitating interactions between FAM134B and LC3B. Additionally, ubiquitinated RHDs strengthen the interactions between adjacent RHDs, forming dense FAM134B clusters that contribute to extensive membrane remodeling 37. Beyond the FAM134B, AMFR also mediates the ubiquitination of the RHD of ARL6IP1, which does not directly interact with LC3. Instead, ARL6IP1 forms a heterodimer with FAM134B, allowing AMFR-mediated ubiquitination of ARL6IP1 to enhance the FAM134B-LC3B interaction, thereby promoting ER-phagy and improving cellular fitness. Defects in ARL6IP1 are associated with sensory nerve loss, abnormal ER morphology, reduced ER stress resistance, impaired ER-phagy, and decreased cellular adaptability 38. Deubiquitination, the reverse of ubiquitination, is mediated by deubiquitinating enzymes that remove Ub molecules from substrate proteins, thereby protecting them from proteasomal degradation. USP20 specifically cleaves K48/K63-linked Ub chains from FAM134B, stabilizing it and enhancing FAM134B-LC3B interactions to facilitate ER-phagy under starvation conditions. Moreover, USP20 interacts with ER TM proteins (VAMP-associated proteins [VAPs]), which recruit autophagy initiation proteins like WD repeat domain and phosphoinositide interacting 2 (WIPI2), enhancing ER-phagy 39.

O-GlcNAcylation, facilitated by O-GlcNAc transferase (OGT), transfers a single N-Acetyl glucosamine to serine or threonine residues, and can be reversed by O-GlcNAc glycosylase (OGA) for de-O-GlcNAcylation. Upregulated O-GlcNAcylation mitigate apoptosis and senescence in nucleus pulposus (NP) cells deprived of nutrients. This protective effect is mediated through the interaction between OGT and FAM134B, which stabilizes FAM134B, reduces FAM134B ubiquitination and degradation, and enhances ER-phagy, thereby facilitating cellular adaptation and survival under nutrient-deficient conditions 40. Nε-lysine acetylation within the ER is another key mechanism regulating ER homeostasis and protein secretion. CBP acetylates and SIRT7 deacetylates FAM134B at K160 site, respectively, creating a regulatory circuit for ER homeostasis. ER stress-induced acetylation of FAM134B by CBP promotes FAM134B oligomerization, ER membrane fragmentation, and ER-phagy. Then, acetylated FAM134B enhances CAMKII-mediated phosphorylation, inducing mild and sustained ER-phagy. Conversely, SIRT7 deacetylates FAM134B to prevent excessive ER degradation. Interestingly, the nuclear acetyltransferase CBP, traditionally recognized as a transcriptional regulator, can relocate to the cytosol to modulate ER stress responses by regulating ER-phagy 41, 42. AT1/SLC33A1 facilitates cytosol-to-ER acetyl-CoA flux, negatively regulates ER-phagy levels by suppressing the ATG9A-FAM134B/SEC62-LC3 axis. Acetylation of ATG9A disrupts its interaction with FAM134B and SEC62, thereby inhibiting ER-phagy. In this process, the chaperone protein CALR binds to acetylated ATG9A within the ER lumen, whereas HSPB1 undergoes a conformational change to bind to ATG9A, further preventing its interaction with FAM134B and SEC62 and ultimately inhibiting ER-phagy. AT-1/SLC33A1 overexpression in mice results in a progeria-like phenotype, characterized by aberrant metabolism and systemic inflammation 43, 44.

### FAM134B in inflammatory and immune diseases

#### Sepsis

Sepsis is a critical syndrome marked by multiorgan dysfunction due to an abnormal host response to infection. Myocardial injury is a frequent complication of sepsis and septic shock, occurring in up to 40% of cases, and is a significant contributor to poor prognosis. In sepsis, FAM134B and ER-phagy is upregulated to serve a protective role by attenuating myocardial injury through the suppression of inflammatory responses and apoptosis. FAM134B overexpression or rapamycin (RAP, an autophagy inducer) treatment significantly reduces myocardial lesions in septic mice, whereas FAM134B knockout or treatment with 3-methyladenine (3-MA, an autophagy inhibitor) reverses these protective effects 45.

#### Viral infectious

**Figure 3:**
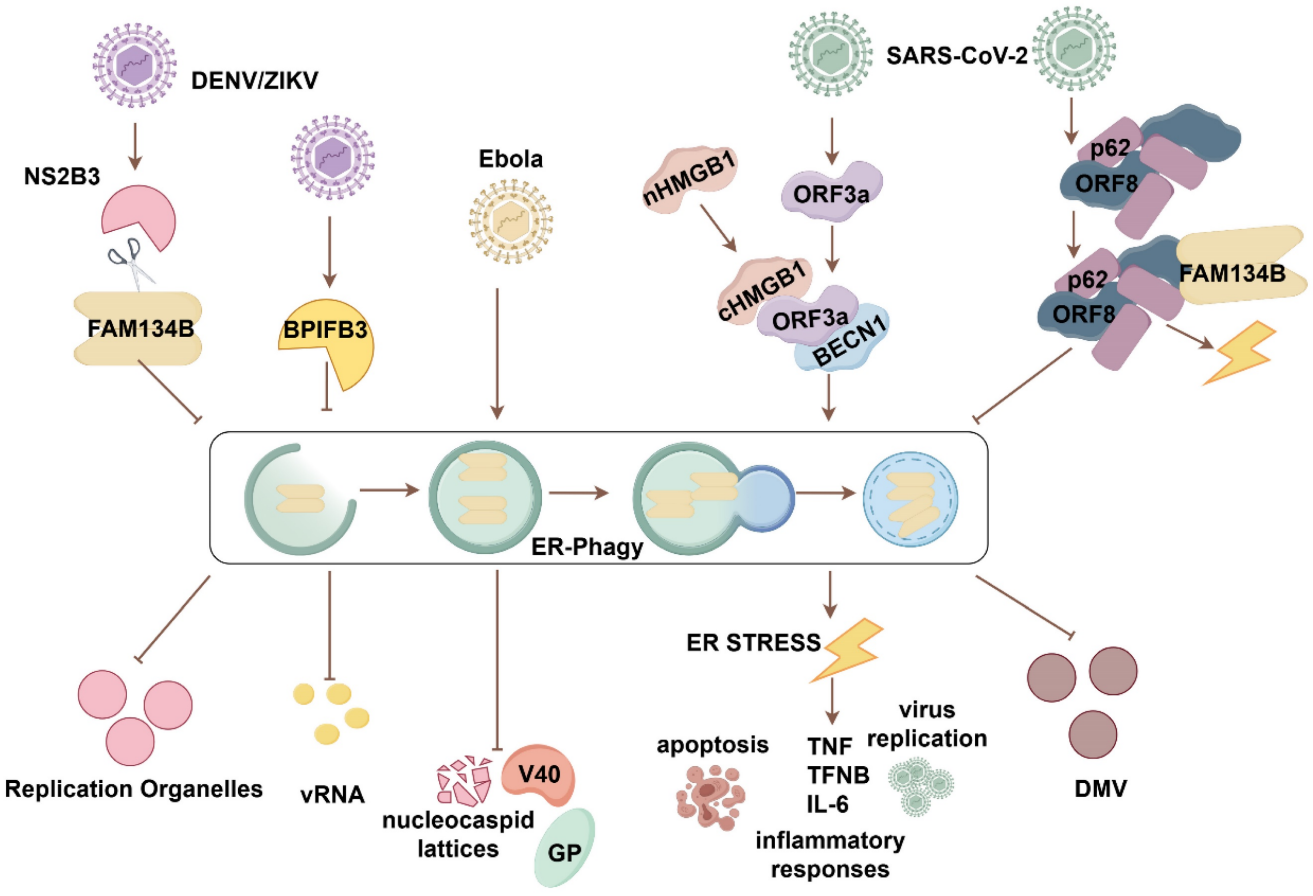
** FAM134B-mediated ER-phagy in viral infectious diseases.** During DENV and ZIKV infection, the cleavage of FAM134B by NS2B at the RHD site disrupts FAM134B oligomerization, impairing ER-phagy and enhancing viral replication. BPIFB3 is an autophagy regulator that positively regulates DENV and ZIKV replication. Inhibition of FAM134B-mediated ER-phagy by BPIFB3 results in increased viral replication. In Ebola virus infection, FAM134B-mediated ER-phagy exerts antiviral effects by suppressing the expression of viral replication-associated proteins GP and VP and reducing the accumulation of nucleocapsid lattices. During COVID-19 infection, SARS-CoV-2 ORF3a manipulates ER-phagy by interacting with HMGB1, facilitating FAM134B-mediated ER-phagy via the HMGB1-BECN1 pathway, leading to ER stress, inflammation, cell death, and viral replication. SARS-CoV-2 ORF8 undergoes phase separation and interacts with p62 to form condensates. These ORF8/p62 condensates hijack FAM134B/ATL, preventing its interaction with LC3 and impairing ER-phagy. This disruption results in increased viral DMV formation and severe ER stress.

The ER provides membranes for virus-replicating organelles, facilitating viral assembly and maturation. Studies have established FAM134B's key role in viral infection pathogenesis (**Fig. 3**). Early in flaviviral infections, FAM134B's antiviral effect against dengue virus (DENV) and Zika viruses (ZIKV), linked to reduced viral RNA production, was confirmed through FAM134B knockdown experiments. The flaviviral protease NS2B3 enhances viral replication by manipulating FAM134B-mediated ER-phagy. Specifically, NS2B3 cleaves the RHD of FAM134B, disrupting its oligomerization and membrane bending, thereby inhibiting ER-phagy and promoting replication vesicle formation 46. The depletion of the flavivirus positive regulator BPI fold-containing family B member 3 (BPIFB3) is hypothesized to increase ER sheet turnover and promote FAM134B-mediated ER-phagy 47. FAM134B contributes to the inhibition of Ebola virus replication, specifically in the Makona and Mayinga strains. Increased levels of virus-associated glycoprotein (GP) and VP40, along with the accumulation of nucleocapsid lattices, were observed in FAM134B^-/-^ cells compared with FAM134B^+/+^ cells 48. Whole-exome sequencing revealed three single nucleotide variants of *CCRL2* (rs3204849), *RETREG1/FAM134B* (rs61733811), and *YWHAH* (rs73884247) in linked to pediatric HIV associated neurocognitive disorder. FAM134B knockdown exacerbates the IL-1β release, and suppresses autophagy in microglia cells upon HIV ssRNA40 exposure 49.

The impairment of FAM134B-mediated ER-phagy has been contributed to the pathogenesis of SARS-CoV-2 infection. In particular, ORF3a protein is capable of subverting FAM134B-mediated ER-phagy, thereby facilitating its own replication and propagation. ORF3a targets the ER, promoting HMGB1 movement from the nucleus to the cytoplasm and its recruitment to the ER. ORF3a interacts with HMGB1 and promotes FAM134B-mediated ER-phagy via the HMGB1-BECN1 pathway. Sustained ER-phagy exacerbates ER stress, early apoptosis, inflammatory cytokine storms, and viral replication. Therefore, proteins involved in ER-phagy and ER stress pathways could be potential therapeutic targets for SARS-CoV-2 infection **50**. Accompanied by hijacking FAM134B and ATL3, ORF8 interacts with P62 to undergo an ORF8/P62 condensates. It subsequently attenuates the antiviral efficacy of FAM134B-mediated ER-phagy, thereby facilitating the formation of double-membrane vesicles (DMVs) that are conducive to viral replication. Importantly, ORF8 homodimer formation is essential for the hijack of FAM134B and ATL3^51^. Blocking ORF8 homodimerization may serve as an effective antiviral strategy to inhibit viral replication.

#### Allergic rhinitis

Genetic analysis identified that the single nucleotide polymorphism (SNP) rs7071836 in the *CD39* gene and rs257174 in the *FAM134B* gene are associated with heightened inflammation and an elevated risk of allergic rhinitis patients. Subsequent analysis demonstrated that reduced CD39 in regulatory T cells impairs the hydrolysis of extracellular ATP. Concurrently, the rs257174 SNP enhances FAM134B expression and further leads aberrant immune response in monocytes 52.

### FAM134B in neurodegenerative diseases

#### Hereditary neuropathies

HSAN2 is a rare autosomal recessive disorder resulting from specific genetic mutations. It is characterized by early-onset impairment of sensory and autonomic nerves, with a predominant impact on distal sensory functions. HSAN2 patients frequently complicated with changes in skin nutrition, recurrent ulcers, soft tissue infections, and osteomyelitis, potentially leading to distal amputation. HSAN2 has four subtypes, with numerous reports linking *FAM134B* mutations to subtype HSAN2B (**Table 2**) 53-58. FAM134B knockdown leads to changes in Golgi apparatus structure and size, along with increased neuronal apoptosis. Notably, the homozygous FAM134B^W107X^ mutation led to chronic renal failure with HSAN2B, indicating that renal failure might be an advanced stage of FAM134B-related diseases. The FAM134B mutation likely causes pathological progression due to autoinflammation from ER-to-Golgi axis disruption 59. Excessive cell death in dorsal root ganglion sensory neurons, caused by the FAM134B^G216R^ mutation, could be attenuated by modulating FAM134B oligomerization levels, highlighting a potential therapeutic target for HSAN2 33.

Epidermal growth factor transactivates NTRK2/TrkB, promoting its transport to the cell surface and augmenting its responsiveness to brain-derived neurotrophic factors, a process essential for the proper development of the cortical plate. ER-phagy degrades NTRK2/TrkB to restrict cell membrane trafficking. CNX directs NTRK2/TrkB to FAM134B, where it undergoes lysosomal degradation. Phosphorylation of CNX releases NTRK2/TrkB from the ER, enabling its cell surface transport. Strikingly, CNX determines whether NTRK2/TrkB is transported to the cell surface or targeted for degradation via autophagy 60-62. In a rat model of prolonged cervical cord compression, FAM134B levels increased following compression and were further elevated in neuronal cells after melatonin treatment. Furthermore, glutamate-induced neurotoxicity resulted in increased FAM134B expression in neuronal cells 63.

#### Protein aggregation disorders

FAM134B-mediated ER-phagy helps degrade the Niemann-Pick type C1 (NPC1) mutant. The NPC1^I1061T^ mutation causes NPC disease, a fatal progressive neurodegenerative disorder. Misfolded NPC1 is also degraded via the E3 ligase MARCH6-dependent ERAD, contributing to the complementary regulation of protein turnover 64. In Parkinson's disease, α-synuclein accumulation is linked to a reduction in dopaminergic neurons and motor dysfunction. Accumulated α-synuclein in the ER binds to CNX and is targeted for clearance via FAM134B-mediated ER-phagy, alleviating ER dysfunction and protecting dopaminergic neurons 65.

#### Age-related degeneration

Accumulated advanced glycation end products disrupts mitochondrial and ER homeostasis by generating ROS. Then, activated ROS pathway upregulates senescence and apoptosis-related proteins (p53, p16, and caspase-3), ultimately contributing to intervertebral disc degeneration (IDD). FAM134B-mediated ER-phagy exerts a protective effect by modulating ROS signaling, thereby reducing apoptosis and senescence in NP cells 66. Moreover, nutrient-deprived IDD tissues and cells exhibit increased proteins associated with apoptosis and senescence, including p53, p21, p16, caspase-3, and Bax. OGT interacts with FAM134B to stabilize the protein, preventing ubiquitination and degradation. OGT overexpression or O-GlcNAcase inhibition enhances FAM134B and ER-phagy, promoting NP cell survival and delaying IDD progression under nutrient-deprived conditions. Therefore, O-GlcNAcylation may represent a potential therapeutic target for IDD disease 40. Caloric restriction has been demonstrated to enhance skeletal muscle health in the elderly population. GEO analysis revealed increased FAM134B expression in aged skeletal muscles subjected to caloric restriction, suggesting that FAM134B-mediated ER-phagy contributes to muscle maintenance and longevity 67.

### FAM134B in cancer

#### Tumor-Suppressive Roles

Reduced *FAM134B* expression promotes colorectal adenoma transition toward colorectal adenocarcinoma, enhances tumor recurrence and metastasis, and reduces patient survival rates 68, 69. Mutations in *FAM134B* induce structural and functional abnormalities, with the most common mutation sites identified in colorectal cancer (CRC) tissues and cells being p.Glu354Glu, p.Val342Ala, p.Thr359Thr, p.Ser276Cys, and p.Asp349ArgfsX13 70. Moreover, copy number variations in *FAM134B* are associated with cancer progression 71. FAM134B expression is progressively downregulated as the disease advances, and its inhibition enhances colon cancer cell proliferation and colony formation. A xenograft model revealed increased tumor formation in FAM134B-deficient mice, further supporting its tumor-suppressive role 72. FAM134B interacts with several proteins in colon cancer cells, including end-binding protein (EB1), adenylyl cyclase-associated protein 1, peptidyl-prolyl cis-trans isomerase B, and ER protein retention receptor 2 (KDELR2). These interactions influence colon carcinogenesis and subcellular structure regulation. FAM134B downregulation decreases KDELR2 expression while increasing EB1 expression. As KDELR2 is potentially involved in protein secretion pathways, its downregulation disrupts cellular homeostasis, contributing to tumorigenesis. EB1 interacts with adenomatous polyposis coli (APC) and aurora kinase B, modulating microtubule-associated processes that promote tumor cell growth. FAM134B knockdown upregulates EB1 expression, which activates the WNT/β-catenin pathway, thereby promoting adenoma-to-adenocarcinoma progression in CRC 73. FAM134B also stabilizes APC and p53 via the WNT/β-catenin pathway and interacts with MIF and p53 to control mitochondrial metabolism and the cell cycle. Its overexpression leads to G1-phase arrest as well as reduced apoptosis and mitochondrial respiration, thereby limiting tumor cell growth 74. FAM134B promoter hypermethylation negatively correlated with FAM134B expression, in good agreement with tumor metastasis and poor prognosis in CRC. In addition, microRNA-186-5p inversely affects FAM134B levels, promoting tumor growth and worsening prognosis. Therefore, FAM134B promoter methylation and microRNA-186-5p retain a promising potential for CRC therapies 75, 76. In addition, FAM134B might suppress tumors in malignant mesothelioma and is considered a potential antigen for an mRNA vaccine, especially aiding patients with the TM2 immune subtype 77.

#### Oncogenic Roles

**Figure 4:**
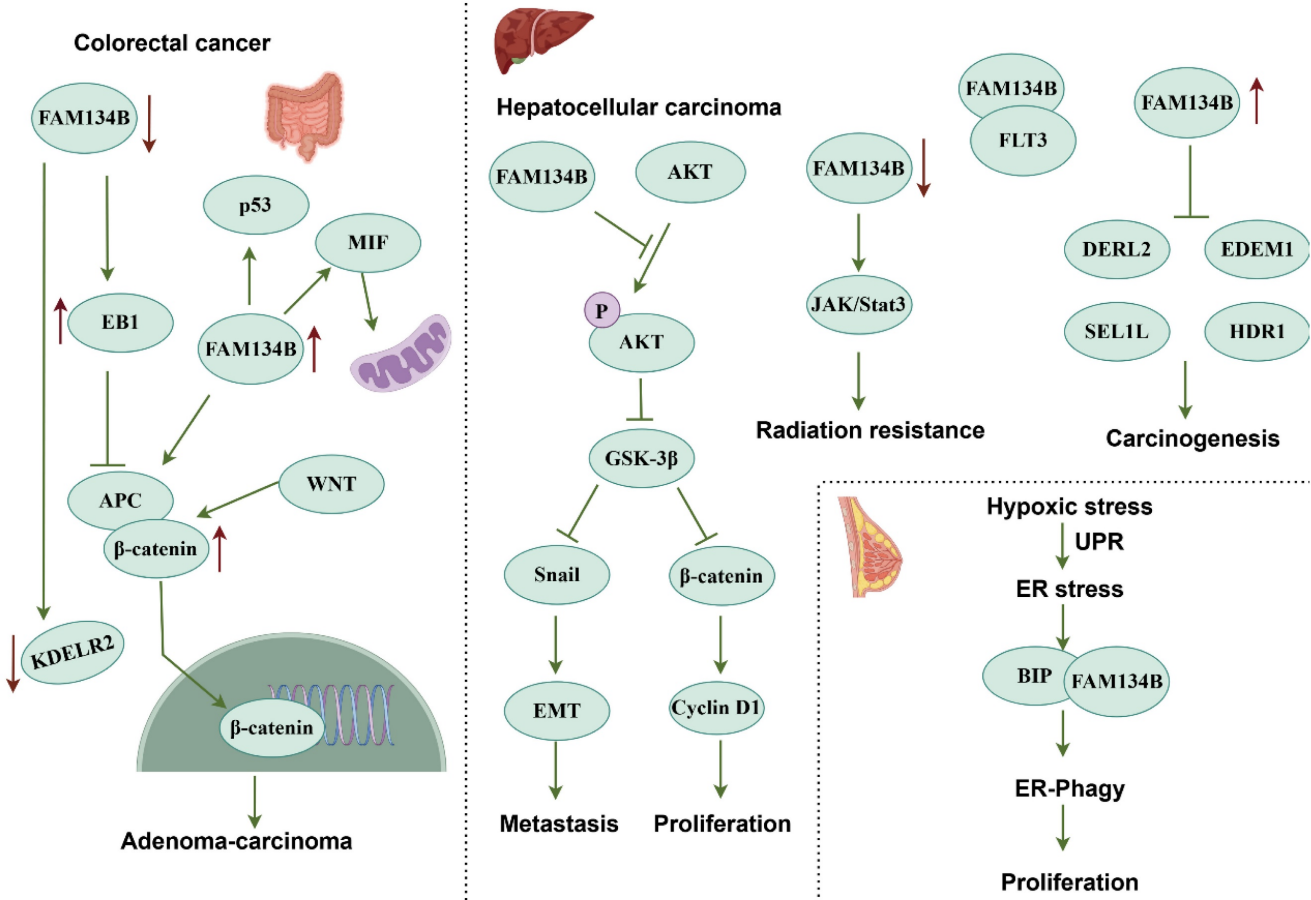
** Signaling pathways of FAM134B in tumor-related diseases.** FAM134B suppresses colorectal cancer by inhibiting EB1 and upregulating KDELR2 expression, which in turn activates the WNT/β-catenin pathway. FAM134B also upregulates MIF and p53 levels via the WNT/β-catenin pathway, influencing cell cycle regulation and mitochondrial function. Conversely, *FAM134B* acts as an oncogene in HCC, promoting tumorigenesis, EMT, and tumor metastasis through the AKT signaling pathway. Downregulation of FAM134B in HCC contributes to radiotherapy resistance by modulating the JAK/STAT3 pathway. Moreover, FAM134B suppresses the expression of ER stress-related degradation factors such as DERL2, EDEM1, SEL1L, and HDR1, thereby accelerating HCC progression.

FAM134B demonstrates a pro-carcinogenic function in esophageal squamous cell carcinoma, as its inhibition leads to a reduction in migration, and invasion 78. Copy number variations and 37 mutation sites of FAM134B have been identified in cancerous tissues, and mutations in FAM134B have been associated with metastatic lymph nodes 79. FAM134B-mediated ER-phagy is also implicated in melanoma tumor stem cell regulation. In M14-SE cells, FAM134B-mediated ER-phagy promotes tumor formation and the tumor stem cell markers, such as Aldh1, CD133, Nanog, Oct4, and Sox2. In addition, SEC23a suppresses tumor stemness gene expression and self-renewal by inhibiting ER stress and FAM134B-mediated ER-phagy 80. *FAM134B* has been identified as a pivotal gene in the integrated diagnostic network used for breast cancer detection 81. Notably, hypoxia represents a major challenge in cancer therapy. Hypoxia-induced ER stress induce misfolded protein accumulation, activating the UPR. BiP chaperones detach to identify these proteins and interact with FAM134B, thereby triggering ER-phagy to clear damaged ER, reduce stress, and maintain homeostasis. Silencing FAM134B increases ER stress, while BiP depletion hinders ER-phagy and cancer cell growth, positioning ER-phagy targeting as a breast cancer therapeutic strategy (**Fig. 4**) 82.

In hepatocellular carcinoma (HCC), FAM134B exerts oncogenic properties by promoting tumorigenesis, epithelial-mesenchymal transition (EMT), and metastasis through AKT signaling pathway. Specifically, FAM134B-induced activation of AKT phosphorylated and inhibited GSK-3β, resulting in β-catenin accumulation, which enhances HCC cell proliferation. FAM134B stabilizes Snail expression, further facilitating EMT 83. The downregulation of FAM134B impairs cell proliferation and triggers apoptosis, suggesting its involvement in HCC progression. Mechanically, FAM134B promotes HCC progression by inhibiting DERL2, EDEM1, SEL1L, and HRD1 84.

Interestingly, FAM134B interacts with FMS-related receptor tyrosine kinase 3 (FLT3) to activate the JAK2/STAT3 pathway, enhancing radiotherapy sensitivity, suggesting its potential as a therapeutic target 85. Moreover, ferroptosis enhancement in HCC is a promising strategy against treatment resistance. The ferroptosis inducer sorafenib activates FAM134B-mediated ER-phagy, whereas inhibition of FAM134B enhances sorafenib-induced ferroptosis in HCC cells. *In vivo*, sorafenib reduced tumor growth and downregulated FAM134B and GPX4, aligning with *in vitro* results. The RNA-binding protein PABPC1 binds and upregulates FAM134B, indicating that PABPC1-FAM134B-ER-phagy pathway targeting might be a viable HCC treatment strategy 86. In Ewing sarcoma, FAM134B-mediated ER-phagy supports dormant cell survival. When the oncogenic fusion gene EWSR1/FLI1 is inhibited, approximately 1% of the cells enter dormancy, depending on this pathway for persistence 87.

### FAM134B in metabolic diseases

**Figure 5:**
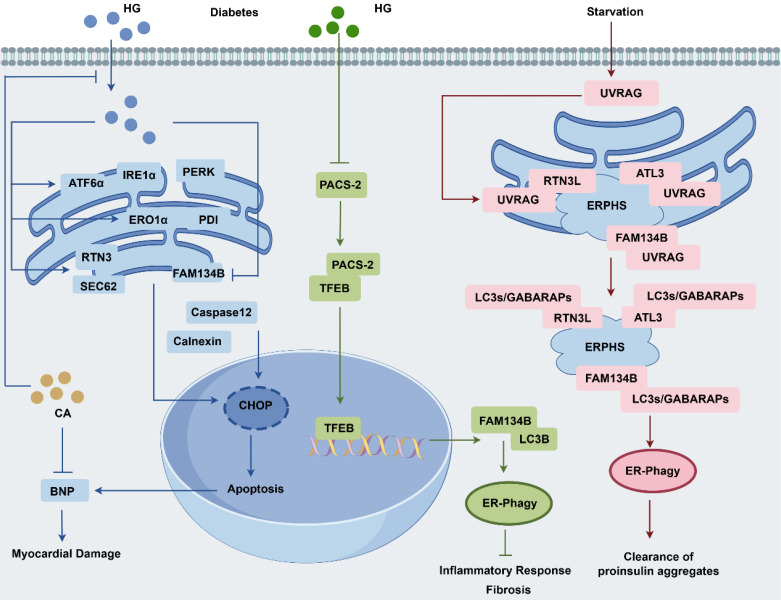
** Regulation of FAM134B in diabetes and proinsulin clearance.** High glucose levels in diabetes induce ER stress, activating ER-phagy pathways associated with RTN3L and SEC62 while simultaneously inhibiting FAM14B, leading to cell death and potential DCM. Chlorogenic acid has been shown to reduce ER stress and cell death in DCM models by upregulating FAM134B expression. In DKD, a reduction in ER-phagy in renal tubular cells is observed, primarily as a result of decreased levels of PACS-2 and FAM134B. PACS-2 plays a crucial role in TFEB nuclear translocation, which subsequently enhances FAM134B expression. The absence of PACS-2 exacerbates kidney damage in DKD by impairing ER-phagy via the TFEB/FAM134B pathway. UVRAG plays a role in proinsulin aggregate clearance, modulating ER-phagy through interactions with cargo receptors, including FAM134B, ATL3, and RTN3L. This process facilitates Atg8 protein recruitment and promotes receptor oligomerization, enhancing the efficient assembly of ER-phagy sites.

In an *in vitro* model of diabetes, high glucose levels triggered ER stress and ER-phagy, and apoptosis, which may contribute to the diabetic cardiomyopathy (DCM) progression **(Fig. 5)**. In HepG2 cells, high glucose exposure induced the upregulation of ER stress and upstream regulatory proteins (GRP78, PERK, IRE1α, ATF6, αPDI, and ERO1α). Concurrently, it also elevated the ER-phagy proteins (Sec62 and RTN3), whereas FAM134B expression was notably decreased. Moreover, CNX and apoptosis-related proteins (CHOP and caspase-12) were upregulated. *In vivo* model, structural abnormalities were observed in the myocardium, and BNP, a marker of myocardial damage, was elevated. In the above model, chlorogenic acid reduces ER stress and cell death by boosting FAM134B to ER-phagy 88. In non-alcoholic fatty liver disease (NAFLD) rats, high-fat diet increased p62 and FAM134B levels and disrupted the ER-phagy, prolonged ER stress, and CHOP, GRP78, and TNF-α upregulation, thereby contributing to precancerous lesions. Notably, the hepatic metabolic enzyme inducer phenobarbital (PB) inhibits precancerous lesion formation by activating ER-phagy and reducing ER stress 89. NAFLD is another common complication of T2DM, characterized by the upregulation of ER stress proteins (GRP78 and ATF6) and downregulation of ER-phagy markers (FAM134B, p62, Beclin-1, and LC3II/I). Chinese herbs 'Jianpi Xiaozhi formula' shows protective effect of liver injury in T2DM rats by modulating ER stress and ER-phagy 90. In a streptozotocin-induced model of diabetic kidney disease (DKD), renal tubular cell dysfunction significantly disrupted cellular homeostasis, leading to inflammatory cell infiltration and interstitial fibrosis. This was mainly linked to decreased PACS-2 expression, which activates the TFEB-FAM134B axis that alleviates inflammatory and fibrotic responses 91. Collectively, boosting FAM134B-mediated ER-phagy in diabetic patients could protect the heart, liver, and kidney, offering a new treatment approach for metabolic diseases.

Ultraviolet (UV) resistance-associated gene (UVRAG), a protein integral to UV radiation resistance, interacts with FAM134B, ATL3, and RTN3L via its N-terminal PR structural domain. The interaction is critical for initiating autophagosome formation at ER-phagy sites, autophagosome maturation and lysosomal fusion. Notably, UVRAG enhances ER-phagy by promoting ER-phagy receptor oligomerization, potentially reducing the cellular accumulation of pathogenic proinsulin 92. Hepatic steatosis is more vulnerable to ischemia/reperfusion injury, which triggers oxidative stress and inflammatory responses in hepatocytes. Interestingly, berberine exerts a protective effect against hepatic steatosis by inhibiting FAM134B-mediated ER-phagy 93. In hepatocytes, dithiothreitol (DDT) induces ER stress, characterized by a temporal increase in CHOP, GPR78, and CNX expression, leading to mitochondrial calcium destabilization and apoptosis. However, DDT-induced ER stress and apoptosis could be reversed by FAM134B-mediated ER-phagy, providing a protective mechanism against cellular damage 94.

FAM134B positively regulated the lipid metabolism and subcutaneous lipid deposition in pigs, largely owing to activating lipogenic genes, including fatty acid synthetase and acetyl-CoA carboxylase, while suppressing adipose triglyceride lipase and hormone-sensitive lipase. Notably, adipocyte differentiation is inhibited in cells treated with pFAM134B siRNA, suggesting that targeting FAM134B may offer a novel approach for controlling adiposity and managing metabolic diseases 95. In acute pancreatitis, ER stress-related proteins like BiP, p-PERK, and p-eIF2α increase, while ATF6 degrades more. High cleaved-caspase-3, p-MLKL, and Rip3 levels lead persistent ER stress and progressed to apoptosis and necrosis. Early on, CCPG1- and FAM134B-mediated ER-phagy boosts pancreatic cell survival, but feedback between ER-phagy and ER stress eventually hinders ER clearance, worsening the condition 96. FAM134B-mediated ER-phagy is essential for maintaining in skeletal muscle homeostasis during acute exercise. Acute exercise reduces SR/ER calcium ATPase activity, causing cytoplasmic Ca^2+^ accumulation and subsequent muscle dysfunction. This is accompanied by increased ER stress proteins (PDI, GRP78, and CRT) and ER-phagy markers (FAM134B and LC3B), which gradually stabilize throughout exercise 97.

### FAM134B in cardiovascular diseases

The rare SNP of *FAM134B* (rs78314670) has been regarded as a potential genetic site of thromboembolism susceptibility. This SNP of *FAM134B* correlates with reduced levels of the anticoagulant glucosylceramide, thereby affecting protein C activation and influencing sphingolipid metabolism 98. A strong epistatic effect between FAM134B and TNFRSF19 has also been observed, highlighting a significant link to vascular dementia predisposition 99. In a doxorubicin-induced cardiotoxicity model, the activation of FAM134B-mediated ER-phagy was enhanced via the caspase-11/GSDMD signaling pathway, leading to increased apoptosis and aggravated myocardial damage. The formation of GSDMD-induced pores in the ER membrane triggered ER stress, which in turn activated general autophagy and FAM134B-mediated ER-phagy, ultimately culminating in cardiomyocyte apoptosis and substantial myocardial injury. Selective silencing of GSDMD in cardiomyocytes preserved cardiac function and alleviated doxorubicin-induced cardiotoxicity by reducing FAM134B-mediated ER-phagy 100. Apelin-13, an endogenous APJ receptor ligand, induces myocardial hypertrophy, characterized by increased cardiomyocyte size, volume, and protein content. The hypertrophic response is mediated by activation of the pannexin-1/eATP/purinergic ligand-gated ion channel 7 (P2X7) axis and FAM134B-mediated ER-phagy. Specifically, apelin-13 stimulates pannexin-1 hemichannels, leading to ATP release and subsequent eATP-mediated P2X7 receptor activation. This process further activates FAM134B-mediated ER-phagy, thereby promoting cardiac hypertrophy 101.

### FAM134B as a biomarker and potential therapeutic target in diseases

The modulation of FAM134B expression and ER-phagy represents a promising therapeutic avenue for disease treatment and pharmacological innovation (**Table 3** and **Table 4**). Vitexin, a potential inhibitor of breast cancer progression, has been found to inhibit BiP-FAM134B complex formation, suppress BiP-dependent UPR and ER-phagy, and reduce neoplastic cell proliferation in mouse breast cancer xenograft models 82. Investigators have developed an electrochemical approach to quantify FAM134B mRNA levels in patients with esophageal cancer. This method involves magnetic isolation of the target mRNA, adsorption onto SPE-Au, and quantification via differential pulse voltammetry using a [Fe (CN)6] 4-/3- redox system. Notably, this PCR-free assay has exhibited high sensitivity in detecting tumor-specific mRNA in esophageal cancer, making it a promising tool for early cancer diagnosis 102. LOP complexes upregulate ATF4, which subsequently induces FAM134B- and TEX264-mediated ER-phagy and autophagic cell death in glioblastoma cells 103. Brefeldin A (BFA), an inhibitor of protein secretion from the ER to the Golgi apparatus, is encapsulated in mesoporous silica nanoparticles (MSNs) to create MSNs-BFA. The combination of MSNs with the autophagy-inducing peptide TAT-B enhances BFA translocation to the perinuclear region, ensuring controlled release. This strategic localization induces a perinuclear ER stress response, leading to ER expansion. BFA deactivates the AKT/TSC/mTOR pathway to increases the LC3II/LC3I ratio and non-selective autophagy. However, it also suppresses the transcriptional regulation of FAM134B, thereby inhibiting ER-phagy. Ideally, MSN-BFA could be optimized to induce non-selective autophagy while preserving ER integrity, preventing ER fragmentation and enhancing cell viability 104.

Regarding disease diagnosis, FAM134B serves as an exosome-specific marker for the quantitative detection of colon cancer. Serum exosomes are first captured using CD9 or CD63 magnetic beads and subsequently identified using FAM134B functionalized with CdSe quantum dot. After magnetic elution and purification, electrochemical methods are employed to measure the amplified Cd^2+^ quantum dot signals, thereby quantifying the exosome contents. This method achieves a detection sensitivity of 100 exosomes/µL. Quantum dots are gaining attention for their applications in bioimaging, medical diagnostics, and therapy; however, biosafety remains a major concern within the scientific community 105. CdTe quantum dots enter renal cells via clathrin-dependent endocytosis, leading to ER swelling and vacuolization. They trigger FAM134B-mediated ER-phagy through the PERK-ATF4 pathway, resulting in reduced cell viability and impaired renal function. Inhibiting the UPR and silencing FAM13B has been shown to mitigate CdTe-induced cytotoxicity 106. In a nutrient-rich environment, hypothalamic cells treated with acute palmitate shows increased ER stress and FAM134B-mediated ER-phagy via the p-PERK-ATF4 pathway. However, chronic palmitate treatment disrupts autophagosome maturation, exacerbating ER stress and impairing ER-phagy. Palmitate shields hypothalamic cells from stress by inhibiting ER-phagy under starvation 107. Similarly, Z36 exposure of Hela cells boosts FAM134B, LC3, and Atg9, causing excessive ER-phagy and subsequent cell death. Moreover, Z36-induced ER stress and UPR activate the PERK-ATF4-CHOP pathway, promoting cell death, whereas the IRE1 pathway contributes to cell survival. Thus, modulating FAM134B expression via Z36 to promote cancer cell death represents a promising strategy for cancer therapy 108.

## Conclusions and prospects

The RHD and LIR motifs of FAM134B are crucial for ER-phagy, with RHD involved in membrane remodeling and LIR in LC3 binding. The LIR motif has co-evolved with autophagy proteins like LC3 to enhance ER-phagy efficiency. Despite variations in the disordered domain, these functions are conserved, coordinating with RHD to adapt to the regulatory needs of different species, e.g., phosphorylation site-related differences. 13. In contrast, the C-terminal region of FAM134B binds the GABARAP subfamily in a “LIR core + C-helix” mode with approximately 10-fold higher affinity than the LC3 subfamily 109. FAM134B collaborates with other ER-phagy receptors to maintain ER homeostasis, with its RHD bending sheet-like ER but the RHD of RTN3L fitting the tubular ER curvature 92. RTN3L and FAM134B can partially compensate functionally during starvation 7. During prolonged ER stress, FAM134B and SEC62 activate at different stages 5. FAM134B2, a truncated variant of FAM134B, helps maintain amino acid balance and cell survival during AAD by the MEF2D-NR4A1-FAM134B2 pathway. Moreover, the regulatory network of transcription factors BACH1 and ZBTB10 with FAM134B2 can be further explored via molecular interaction or functional rescue assessment 17.

FAM134B-mediated ER-phagy exerts distinct effects depending on the specific pathophysiological state. For example, ZIKV blocks ER-phagy to form replication compartments, whereas SARS-CoV-2 disrupts it to create double-membrane replication vesicles 46, 51. It suppresses CRC via the WNT pathway and enhances tumor cell survival in HCC through the AKT pathway. In HSAN2B, FAM134B mutations impair ER-Golgi trafficking, causing cell death, while in melanoma stem cells, FAM134B-mediated ER-phagy boosts stemness markers, aiding tumor survival. FAM134B supports nociceptor and neuron survival within the autonomic ganglia. However, excessive ER-phagy might induce neuronal cell death. In the heart, FAM134B-mediated ER-phagy protects against septic myocardial injury by reducing apoptosis and inflammation 45, although it also contributes to apelin13-induced cardiomyocyte hypertrophy 101. While no clinical trials specifically target FAM134, adjusting its expression holds promising potential for future research. In ER storage disorders, the XBP1-Sestrin2-TFEB/FAM134B pathway facilitates ER-phagy to remove misfolded proteins. The drugs Fluphenazine (FPZ) and Tetrandrine (TET) boost ER-phagy by inhibiting mTORC1 localization to the lysosomes and activating TFEB/FAM134B, without inducing ER stress 110. Thiamet G (TMG), an O-GlcNAcase inhibitor, boosts FAM134B expression both *in vitro* and *in vivo*, helping to alleviate IDD 40. Notably, TMG also reduces pathological tau aggregation in Alzheimer's disease 111.

FAM134B is particularly implicated in inter-organelle crosstalk, especially in Ca^2+^ exchange between the ER and mitochondria^112^. ER-Phagy is crucial for ER quality control, as it removes damaged ER to maintain cellular balance. As technology advances and we understand better FAM134B-mediated ER-phagy, its therapeutic potential will become clearer.

## Figures and Tables

**Table 1:** FAM134B regulatory factors, mechanisms, and physiological roles.

Pathway/Key Factors	Effect on FAM134B	Pathophysiological model	Outcome	References
TFEB/TFE3	Upregulation	Prolonged starvation	Activates ER-phagy, maintains cellular homeostasis	26
YTHDF2	Downregulation	Porcine adipocytes	Reduces fat deposition	31
CAMK2	S141 phosphorylation enhances oligomerization.	ER stress	Maintain cellular homeostasis	33
CK2	Activating phosphorylation-based ubiquitination of FAM134B and FAM134C	mTOR inhibition	Triggers ER-phagy via FAM134B/FAM134C nanocluster formation	35
AMFR	Ubiquitination of FAM134B and ARL6IP1 enhances oligomerization and membrane curvature	​HeLa	Boosts FAM134B-LC3B interaction and ER-phagy;	37-38
USP20	Stabilizes FAM134B by deubiquitination	Starvation	Enhances FAM134B-LC3B interaction and recruits WIPI2 to promote autophagy initiation	39
OGT	O-GlcNAcylation of FAM134B	Nutrient-deprived NP cells	Suppresses apoptosis/senescence and enhances ER-phagy	40
CBP/SIRT7	K160 acetylation/deacetylation of FAM134B	ER stress	Enhances ER-phagy/Prevents excessive ER degradation	41-42
AT-1	Acetylation ATG9A inhibit FAM134B/SEC62-LC3B axis	AT-1-overexpressing mice	ATG9A acetylation blocks ER-phagy, causing progeria-like symptoms; ATase inhibition rescues aging defects	43-44

**Table 2:** Mutations in *FAM134B* causing HSAN2B

Patient	Exon/Intron	cDNA change	Amino acid change	Mutation type	Zygosity	Reference
1	EX2	c.471C > T	p.Q145X	Nonsense	Hom	58
2	EX6	c.926C > G	p.S309X	Nonsense	Hom	56
3	EX2	c.433C > T	p.Q145X	Nonsense	Hom	
4	EX1	c.17-18delCT	p.P7GfsX133	Frameshift	Hom	
5	Intron7	c.873+2T > C	/	Splice site	Hom	
6	EX4	c.646G > A	p.G216R	SNP	Hom	54
7	EX1	c.321G > A	p.W107X	Nonsense	Hom	59
8	EX5	c.826delA	p.W276Vfs*8	Deletion	Hom	55
9	EX6	c.896-897delAA	p.K299Rfs*6	Deletion	Hom	53
10	EX7	c.1426del	p.Q476Rfs*57	Deletion	Hom	
11	EX7	c.1009C > C/T	p.R337*	Nonsense	Het	57
	EX1	c.259_266del	p.L87Efs*51	Frameshift	Het	

* EX: exon; Hom: homozygous; Het: heterozygous.

**Table 3:** FAM134B as a biomarker and potential treatment target for diseases

Reagent	Targeted protein or pathway	Desired outcome	Target indication	Effect on FAM134B	Reference
Vitexin	Inhibits BiP-FAM134B complexes and UPR	Reduces cell proliferation; tumor suppression	Breast cancer	Suppresses	82
Phenobarbital	Relieves ER Stress	Inhibits precancerous lesion formation	NAFLD	Induces	89
Sorafenib	PABPC1-FAM134B-ER-phagy	Reduces tumor weight and growth; tumor suppression	Hepatocellular carcinoma	Induces	86
Chlorogenic acid	PERK, IRE1α, ATF6α, PDI, ERO1α, BNP	Reduces ER stress and cell death	T2DM	Induces	88
LOP complexes	Upregulates ATF4, DDIT3, HSPA5, and p-EIF2A	Induces autophagic cell death	Glioblastoma	Induces	103
MSNs-BFA	Induces ER stress, inactivates AKT/TSC/mTOR, and enhances macroautophagy	Promotes cell survival	U2OS and A549 cells	Suppresses	104
CdTe-QDs	Activates the PERK-ATF4 pathway	Decreases cell viability	Renal dysfunction	Induces	106
Palmitate (nutrient-rich)	p-PERK-ATF4 pathway	Induces ER stress	Hypothalamus	Induces	107
Palmitate (starvation)	Enhances Bcl-2 expression and inhibits ER stress	Relieves cellular stress		Suppresses	
Z36	PERK-ATF4-CHOP/IRE1 pathway	Induces cell death	Hela cells	Induces	108

**Table 4:** The pathological role and targeting of FAM134B-mediated ER-Phagy in diseases

Disease Category	Disease/Condition	Primary Organ/System	Key Cell Type(s)	​​Animal/Experimental Model	References
**Sepsis**	Septic myocardial injury	Myocardium	Cardiomyocytes	Lipopolysaccharide (LPS)-induced cardiomyocytes/Cecal ligation and puncture (CLP) treatment	45
**Viral Infections**	Flaviviruses	Systemic infection	Human brain microvascular endothelial cells	DENV/ZIKV infection	46-47
	Ebola virus (EBOV)	Systemic infection	Mouse embryonic fibroblasts	EBOV-infected Vero E6 cells	48
	SARS-CoV-2 (COVID-19)	Respiratory system	Lung epithelial cells (A549)	A549; HEK293T	50
	SARS-CoV-2	Respiratory system	Vero E6 cell	Vero-E6; HeLa, HEK293T; A549	51
**Neurodegenerative Diseases**	HSAN2B	Autonomic nervous system	Sensory and autonomic ganglion neurons	Fam134b KO mice	54,57
	Niemann-Pick type C disease	Cerebellum, liver	Primary human fibroblasts	Npc1-I1061T mice	64
	Parkinson's disease	Autonomic system	Dopaminergic neurons	AAV-ER-α-syn mouse model	65
**Age-Related Degeneration**	Intervertebral disc degeneration	Spine system	Nucleus pulposus (NP) cells	Annulus Fibrosus Injury rat	40
**Cancer**	Colon cancer	Digestive system	SW-480, SW-48, HCT116	Severe combined immunodeficiency (SCID) mice	73
	Breast cancer	Breast cancer tissue	MCF-7 Cells	Mouse xenograft (hypoxia model)	82
	Hepatocellular carcinoma	liver	HCC tissue	HCC xenograft mice	83
**Metabolic Diseases**	Diabetic cardiomyopathy (DCM)	heart	Cardiomyocytes	HFFD-induced rats + STZ	88
	Non-alcoholic fatty liver	liver	Hepatocytes	HFD-fed rat	89
	Diabetic kidney disease	Kidney (proximal tubule)	Tubular epithelial cells	STZ-induced mice	91
	Acute pancreatitis (AP)	Pancreas	Pancreatic acinar cells	L-arginine-induced mice	96
**Cardiovascular Disease**	Doxorubicin-Induced Cardiotoxicity (DIC)	Heart	Cardiomyocytes	Mice (WT; GSDMD-KO; GSDMD-CKO)	100
	Cardiac hypertrophy	Heart	Cardiomyocytes	HL-1 and H9c2 cell line	101

## Abbreviations

AAT: alpha-1-antitrypsin
AAD: amino acid deprivation
AMFR: autocrine motility factor receptor
ATL: atlastin
APC: adenomatous polyposis coli
BPIFB3: BPI fold-containing family B member 3
BFA: brefeldin A
CNX: calnexin
CK2: casein kinase 2
CRC: colorectal cancer
DCM: diabetic cardiomyopathy
DDT: dithiothreitol
DENV: dengue virus
DKD: diabetic kidney disease
DMV: double-membrane vesicle
ERLAD: ER-to-lysosome-associated degradation
ERAD: ER-associated degradation
EB1: end-binding protein
EMT: epithelial-mesenchymal transition
FLT3: FMS-related receptor tyrosine kinase 3
GP: glycoprotein
HSAN2B: hereditary sensory and autonomic neuropathy type IIB
HCC: hepatocellular carcinoma
IMM: inner mitochondrial membrane
IDD: intervertebral disc degeneration
KDELR2: ER protein retention receptor 2
LIR: LC3-interacting region
MSNs: mesoporous silica nanoparticles
NPC1: Niemann-Pick type C1
m6A: N6-methyladenosine
NP: nucleus pulposus
NCI: neurocognitive impairment
NAFLD: non-alcoholic fatty liver disease
OGT: O-GlcNAc transferase
OGA: O-GlcNAc glycosylase
P2X7: purinergic ligand-gated ion channel 7
RAP: rapamycin
RHD: reticulon homology domain
SNP: single nucleotide polymorphism
TM: transmembrane
T2DM: type 2 diabetes mellitus
Ub: ubiquitin
UV: ultraviolet
UVRAG: UV resistance-associated gene
VAPs: VAMP-associated proteins
WIPI2: WD repeat domain and phosphoinositide interacting 2
YTHDF2: YTH m6A RNA-binding protein 2
ZIKV: Zika virus
3-MA: 3-methyladenine
